# An Online Tool for Correcting Performance Measures of Electronic Phenotyping Algorithms for Verification Bias

**DOI:** 10.1055/a-2402-5937

**Published:** 2024-12-27

**Authors:** Ajay Bhasin, Sue Bielinski, Abel N. Kho, Nicholas Larson, Laura J. Rasmussen-Torvik

**Affiliations:** 1 Northwestern University Feinberg School of Medicine, Chicago, United States

**Keywords:** data analysis, evaluation, system improvement, statistical methods, data collection

## Abstract

**Objectives** Computable or electronic phenotypes of patient conditions are becoming more commonplace in quality improvement and clinical research. During phenotyping algorithm validation, standard classification performance measures (i.e., sensitivity, specificity, positive predictive value, negative predictive value, and accuracy) are often employed. When validation is performed on a randomly sampled patient population, direct estimates of these measures are valid. However, studies will commonly sample patients conditional on the algorithm result prior to validation, leading to a form of bias known as verification bias.

**Methods** We illustrate validation study sampling design and naïve and bias-corrected validation performance through both a concrete example (1,000 cases, 100 noncases, 1:1 sampling on predicted status) and a more thorough simulation study under varied realistic scenarios. We additionally describe the development of a free web calculator to adjust estimates for people validating phenotyping algorithms.

**Results** In our illustrative example, naïve performance estimates corresponded to 0.942 sensitivity, 0.979 specificity, and 0.960 accuracy; these contrast proper estimates of 0.620 sensitivity, 0.999 specificity, and 0.944 accuracy after adjusting for verification bias using our free calculator. Our simulation results demonstrate increasing positive bias for sensitivity and negative bias for specificity as the disease prevalence approaches zero, with decreasing positive predictive value moderately exacerbating these biases.

**Conclusion** Novel computable phenotypes of patient conditions must account for verification bias when calculating performance measures of the algorithm. The performance measures may vary significantly based on disease prevalence in the source population so use of a free web calculator to adjust these measures is desirable.

## Background and Significance

Computable phenotypes of patient conditions are becoming more commonplace in quality improvement and clinical research. 1 These phenotypes are algorithmically derived from data sources such as electronic health record (EHR), insurance claims, or Centers for Medicare and Medicaid Services data and can empower research and improve patient care. 2 3 Algorithm performance measures, such as sensitivity, specificity, and positive and negative predictive values (PPV and NPV) are common measures of validity obtained by comparing the algorithm result to a “gold standard” (e.g., manual chart review). A common validation study design strategy when the condition of interest has low prevalence is to sample based on the algorithm result (e.g., 50 predicted cases and 50 predicted noncases). 4 5 This strategy is both cost-effective and statistically efficient by enriching for likely true positives and improving the expected precision of positive-class performance measures (e.g., sensitivity, PPV). However, this sampling strategy also results in a form of selection bias known as verification bias, which is commonly encountered in diagnostic test evaluation. 6 7 8 Under these conditions, estimates of sensitivity, specificity, and accuracy can be biased, if the sampling design is not taken into consideration. After repeatedly noting the effects of verification bias on our own phenotype validation efforts and those of our collaborators, we endeavored to develop a tool to easily correct for this bias in phenotype validation. Herein, we illustrate the effects of verification bias on performance estimation through an example validation study and develop a user-friendly online tool to facilitate adjustment of performance measures under these validation study scenarios.

## Methods

### Validation Study Sampling Design

Given that EHR-based phenotyping algorithms can be prone to error, it is often of interest to characterize classification performance relative to ground truth based on manual chart abstraction. For phenotyping algorithms, the total number of patients with computable classification results tends to be very large due to ease of implementation (e.g., the entire patient population at a medical institution). Given the potential laborious nature of chart review, algorithm validation studies are often performed on a relatively small subset of the total population. When the expected prevalence of the disease condition is low (i.e., less than 10%), validation studies may have correspondingly low precision for estimating sensitivity and PPV, if patients are randomly sampled from the population. For example, for a disease with prevalence of 2%, in a random sample of 500 patients we expect 10 positive disease patients, on average. Even at a true algorithm sensitivity of 90% (i.e., 9/10 cases correctly identified), the Wilson score 95% confidence interval (CI) would be [0.596, 0.995]. In contrast, 90% specificity would correspond to a 95% CI of [0.870, 0.925]. This disparity in precision can be mitigated by oversampling subjects predicted by the algorithm as a positive case (e.g., 1:1 sampling based on predicted disease status), leading to a more balanced representation of true disease cases and unaffected noncases within the validation sample.

**Fig. 1:**
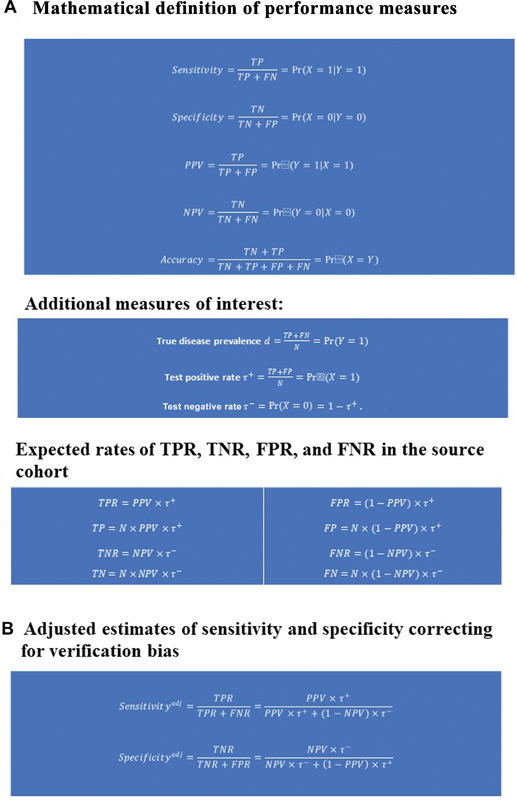
Consider a phenotyping algorithm for predicting the presence of a given disease condition based on a patient's EHR data. We designate *Y* ϵ{0.1} to be the true underlying disease status for a given patient and *Y* ϵ{0.1} to be the predicted disease status by the algorithm, such that 0 and 1, respectively, denote unaffected and affected disease statuses. For disease phenotyping on a patient cohort of size *N* , the classification results can be summarized using a standard 2 × 2 contingency table, which tabulates patient classifications of disease relative to true disease status into four distinct categories: true positives (TP), true negatives (TN), false positives (FP), and false negatives (FN), as indicated in Table 1 . Counts in the equations above can be replaced by corresponding rates by simply factoring out *N* (e.g., the true positive rate  ). Given that unbiased estimates of test positive and negative rates, tau τ ^+^ and τ ^–^ , are available from the algorithm classifications for the original source cohort, the expected rates of TPR, TNR, FPR, and FNR in the source cohort can actually be calculated as simple functions of these parameters and the PPV and NPV estimates from the validation study. For example, recall from above that TPR can be framed as the joint probability Pr( *Y* = 1 *, X* = 1). Since Pr( *Y* = 1 *, X* = 1)= Pr( *Y* = 1 *| X* = 1) **×** Pr ( *X =* 1) by basic rules of conditional probability, and Pr( *Y* = 1|X 1) = PPV ) and Pr( *X* = 1) = τ ^+^ per our definitions above, it follows that *TPR = PPV* **×** τ ^+^ . EHR, electronic health record; FNR, false negative rate; FPR, false positive rate; NPV, negative predictive value; PPV, positive predictive value; TNR, true negative rate; TPR, true positive rate.

While the sampling strategy defined above leads to more statistically efficient estimation of algorithm performance, sampling patients for the validation study based on algorithm-classified disease status can lead to a biased estimation of performance measures. Referred to as “verification” or “workup” bias, unadjusted analyses of the resulting validation 2 × 2 contingency table can specifically lead to overestimated sensitivity while simultaneously underestimating specificity. However, since NPV and PPV correspond to probabilities conditional on predicted statuses, these estimates remain valid under this conditional sampling scheme. Formulae for defining these performance measures correcting estimates of sensitivity and specificity for verification bias are available in Fig. 1 . Detailed explanations of these derivations, along with formulae for calculating corresponding asymptotic CIs, are provided by Begg and Greenes. 9

**Table 1:** 2 × 2 contingency table definitions for phenotyping validation

	Validation study			Source population		
	Chart (+)	Chart (−)	Total	Disease	No disease	Total
Algorithm (+)	49	1	50	98	2	100
Algorithm (−)	3	47	50	60	940	1,000
Total	52	48	100	158	942	1,100

### Illustrative Example

Consider the illustrative example of a validation study where a phenotyping algorithm is applied to a source population of 1,100 patients, corresponding to 100 patients classified as positive and 1,000 patients as negative. From this cohort, 50 predicted cases and 50 predicted noncases were selected for phenotyping algorithm validation. The manual abstraction yielded a 2 × 2 contingency table with counts of 49 true positives, 1 false positive, 3 false negatives, and 47 true negatives.

### Simulation Analysis

To further demonstrate the impact of verification bias on naïve sensitivity and specificity estimates across a broad range of realistic study conditions, we conducted a simple simulation study for a disease with estimated true prevalence between 1 and 50%; true NPV of 0.90, 0.95, and 0.99; and true PPV of 0.70, 0.80, and 0.90. For validation, we considered a balanced study design, such that equal numbers of predicted cases and noncases are selected for chart abstraction. We then calculated the expected bias of naive estimates of sensitivity and specificity relative to appropriately adjusted estimates based on expected values of true positive rate, false positive rate, true negative rate, and false negative rate in the validation study.

### Online Tool

We used Microsoft Visual Studio Code (version 1.78.0) and Python (version 3.10) with the Streamlit package (version 1.13.0) to create a simple tool to calculate sensitivity, specificity, PPV, NPV, and accuracy of a phenotyping algorithm based on chart validation. This can be used for any disease or dichotomous outcome and for validation in multiple situations (not just for EHR) when the prevalence of disease in the validation cohort doesn't match the prevalence of disease in the source cohort. This internet-based tool uses the formulae depicted in Fig. 1 to correct algorithm performance measures based on input values from the validation study and source population. The tool is freely available at: https://bit.ly/3tMTJiE .

## Results

The 2 × 2 contingency table of the example validation study along with projected counts from the total source cohort are presented in Table 1 , whereas respective performance measure analyses corresponding to unadjusted and verification bias-adjusted estimates are presented in Table 2 . Unadjusted performance estimates for the hypothesized phenotyping algorithm corresponded to 0.942 sensitivity, 0.979 specificity, and 0.960 accuracy. The disease prevalence in the validation study sample was 0.520, whereas the true prevalence in the source population was 0.091. After adjusting for verification bias, the updated performance measures for the algorithm corresponded to 0.620 sensitivity, 0.999 specificity, and 0.944 accuracy.

**Table 2:** Comparison of classification performance measures based on unadjusted analysis of the validation study table and verification bias-adjusted estimates

Measures	Naïve		Bias-adjusted	
Prevalence	0.520		0.091	
Accuracy	0.960		0.944	
PPV (95% CI)	0.980	[0.895, 0.999]	–	–
NPV (95% CI)	0.940	[0.838, 0.979]	–	–
Sensitivity (95% CI)	0.942	[0.844, 0.980]	0.620	[0.553, 0.683]
Specificity (95% CI)	0.979	[0.891, 0.999]	0.998	[0.997, 0.998]

Abbreviations: CI, confidence interval; NPV, negative predictive value; PPV, positive predictive value.

Note: PPV and NPV are identical across both analyses.

**Fig. 2:**
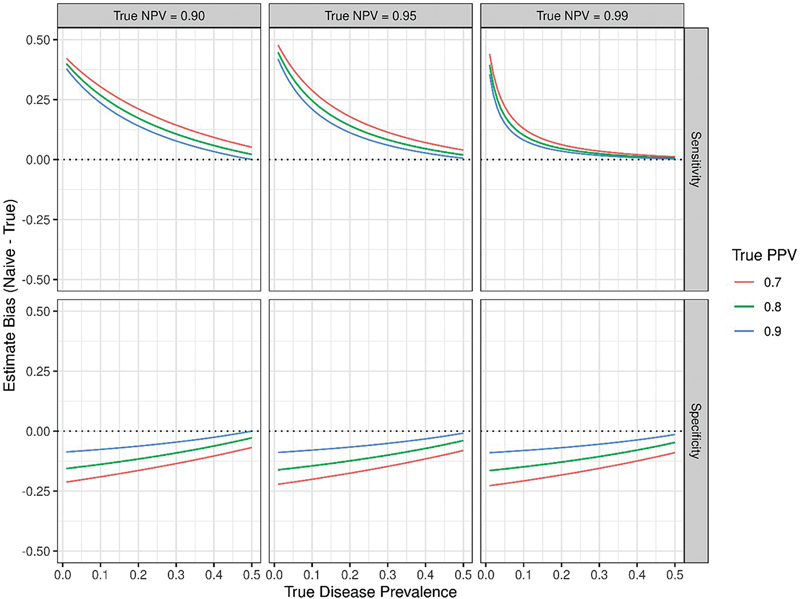
Simulation study results demonstrating expected biases for sensitivity and specificity under verification bias for various values of true PPV, true NPV, and disease prevalence. NPV, negative predictive value; PPV, positive predictive value.

Results from our simulation study are presented in Fig. 2 . These results illustrate the substantial positive bias for sensitivity estimation that may be observed as disease prevalence decreases toward zero when analyzing the unadjusted validation study confusion matrix results. This bias relationship is attenuated as the NPV approaches 1.00, but still yields extreme bias at lower prevalence values. For specificity ( Fig. 2B ), we observe similar trends of increased absolute bias with decreased prevalence. However, the magnitude of this bias remains largely consistent across realistically high values of NPV considered for the simulation study, with lower PPV leading to moderate increases in bias. Of note, these results represented expected biases, and actual results may vary based on sizes of the total population and sampling cohort due to sampling variability.

## Discussion

We have illustrated how much the estimated performance metrics of an algorithm can differ when one does not randomly sample from the source population for algorithm validation. Oversampling of algorithm-positive cases for validation can bias model performance measures, leading to inflated sensitivity and accuracy estimates. The bias can be mitigated by considering the prevalence of disease in the source population and adjusting the calculations to account for the difference.

While sampling conditional on predicted disease status will lead to valid direct estimates of PPV and NPV, these measures are themselves a function of disease prevalence. Thus, they are not necessarily intrinsic properties of a phenotyping algorithm and should be interpreted with caution as disease prevalence may vary across validation populations. 10 Likewise, alternative performance measures that are in part functions of sensitivity and/or specificity, such as F1-score and positive/negative likelihood ratios, will also likely be biased and require similar corrections. Stratified study designs can also be adopted when there are covariates that may correlate with differential algorithm performance, and we refer the reader to appropriate references for how to address adjustment under these conditions. 6 9

For the best adjustment and algorithm calibration, one should undertake the theoretical exercise of defining the source population prior to application of an algorithm. Ideally, a very high percentage of the theoretical source population will have estimated phenotypes generated by the algorithm to obtain precise estimates of the test positive and negative rates; if a high percentage of patients are not classified as either disease positive or negative by the algorithm, then the performance metrics of the algorithm will be more difficult to interpret and researcher should keep in mind the impacts of this on of cross-institutional validation. 11 12 13

This tool will enable clinicians, informaticists, and data scientists to appropriately characterize performance of computable phenotype algorithms. Future extensions of our online tool may include support for complex stratified sampling, calculation of corresponding 95% CIs, and providing support for sample size and power calculations for planning of validation studies.

## Clinical Relevance statement

This tool corrects phenotyping validation algorithms to appropriately reflect phenotyping performance. Valid phenotypes are necessary in research in order for results to be applicable for downstream clinical use.

## Multiple Choice Questions

1. Verification bias:
  - is a form of selection bias
  - verifies study results
  - can create artificially extended survival intervals
  - is not problematic for diagnostic algorithms
  **Answer:** The correct answer is option a. Verification bias occurs when an enriched sample is used to calculate performance measures without considering or adjusting for sampling design.
2. Verification bias may occur when:
  - oversampling positives in a low prevalence cohort for algorithm validation
  - random sampling in a cohort for algorithm validation
  - sampling positives and negatives at the cohort prevalence for algorithm validation
  - randomly sampling population-level data
  **Answer:** The correct answer is option a. Oversampling positives in a low prevalence cohort is an example of enriching the sample for positives, which will result in verification bias and make inaccurate performance measures without considering or adjusting for sample design and true prevalence of condition.
3. Verification bias may:
  - only affects sensitivity
  - only affects specificity
  - affects both sensitivity and specificity
  - affects disease prevalence
  **Answer:** The correct answer is option c. Depending on the prevalence of disease in the source population, the measures of algorithm performance (e.g., sensitivity and specificity) can be over or estimated. The source population must be accounted for in order to provide accurate estimates of model performance.
