# Corrigendum: An Online Tool for Correcting Performance Measures of Electronic Phenotyping Algorithms for Verification Bias

**DOI:** 10.1055/s-0045-1809611

**Published:** 2025-06-26

**Authors:** Ajay Bhasin, Suzette J. Bielinski, Abel N. Kho, Nicholas B. Larson, Laura Rasmussen-Torvik

**Affiliations:** 1 Department of Medicine, Division of Hospital Medicine, Northwestern University Feinberg School of Medicine, Chicago, Illinois, United States; 2 Department of Pediatrics, Division of Hospital-Based Medicine, Northwestern University Feinberg School of Medicine, Chicago, Illinois, United States; 3 Division of Epidemiology, Department of Quantitative Health Sciences, Mayo Clinic College of Medicine and Science, Rochester, Minnesota, United States; 4 Center for Health Information Partnerships, Institute for Public Health & Medicine, Feinberg School of Medicine, Northwestern University, Chicago, Illinois, United States; 5 Division of General Internal Medicine, Department of Medicine, Feinberg School of Medicine, Northwestern University, Chicago, Illinois, United States; 6 Division of Clinical Trials and Biostatistics, Department of Quantitative Health Sciences, Mayo Clinic College of Medicine and Science, Rochester, Minnesota, United States; 7 Department of Preventive Medicine, Division of Epidemiology, Northwestern University Feinberg School of Medicine, Chicago, Illinois, United States

The authors would like to update their affiliations in the above article published in *Applied Clinical Informatics Open* , Volume 8, Number 2, e89-e93, on December 27, 2024 (DOI: 10.1055/a-2402-5937 ) The corrected affiliations appear as above.

